# Preschool morphological training produces long-term improvements in reading comprehension

**DOI:** 10.1007/s11145-016-9636-x

**Published:** 2016-03-17

**Authors:** Solveig-Alma Halaas Lyster, Arne Olav Lervåg, Charles Hulme

**Affiliations:** University of Oslo, Oslo, Norway; University College London, London, UK

**Keywords:** Morphological awareness training, Reading comprehension, Follow-up study, Preschool intervention, Intervention effect

## Abstract

We evaluated the effect of morphological awareness training delivered in preschool (8 months before school entry) on reading ability at the end of grade 1 and 5 years later (in Grade 6). In preschool, one group of children received morphological awareness training, while a second group received phonological awareness training. A control group followed the ordinary preschool curriculum. The comparison between each training condition and the control condition is quasi experimental, whereas the comparison between the morphological and phonological treatments is randomized at group level. In Grade 1 children in the morphological awareness training group had significantly higher scores than children in the control group on both word reading and text reading measures, but no differences were found between the experimental groups. In Grade 6 children in the morphological awareness training group had significantly higher scores compared with the control group on a latent measure of reading comprehension, whereas the children in the phonological awareness training group did not differ from the controls; although the experimental groups did not differ significantly from each other. The results suggest that early training in morphological awareness can have long-term effects on children’s literacy skills.

## Introduction

Morphological knowledge and morphological awareness are terms used to characterise our knowledge and awareness of the morphological structure of words. Morphological awareness is defined as the ability to reflect on and manipulate morphemes, which are the smallest meaning-based elements of words. The association between morphological awareness and reading ability has long been recognised (e.g., Brittain, 1970; Carlisle, 2000; Deacon & Kirby, 2004; Mahony, Singson, & Mann, 2000; Nunes, Bryant, & Bindman, 1997; Tyler & Nagy 1990) and appears to be present in a range of orthographies (e.g., Casalis & Louis-Alexandre, 2000; Elbro & Arnbak, 1996; Kieffer & Lesaux, 2008; Kuo & Anderson, 2006; Lyster, 2010; McBride-Chang, Shu, Zhou, Wat, & Wagner, 2003; Nagy, Berninger, & Abbott, 2006; Sénéchal, 2000; Saiegh-Haddad & Geva, 2008). Morphological awareness is a predictor of individual differences in reading and spelling accuracy (Deacon & Bryant, 2006; Deacon & Kirby, 2004; Kirby et al., 2012; Lee, 2011; Mahony et al., 2000; Nunes, Bryant, & Bindman, 2006), reading comprehension (Cunningham & Carroll, 2013; Deacon & Kirby, 2004; Kirby et al., 2012; Lee, 2011) and vocabulary knowledge (Kieffer & Lesaux, 2012a, b; Wagner, Muse, & Tannenbaum, 2007).

Previous studies do not distinguish clearly between the concepts of morphological knowledge and morphological awareness (Carlisle & Goodwin, 2013). Studies have used different morphological tasks that place different demands on children’s morphological abilities, and it is often difficult to determine whether tests assess tacit or more explicit knowledge. Accordingly, some authors currently use the superordinate concept of morphological knowledge to circumvent the difficulty of distinguishing between the concepts (Bowers, Kirby, & Deacon, 2010; Nagy, Carlisle, & Goodwin, 2014). Here the terms ‘morphological knowledge’ and ‘morphological awareness’ will be used interchangeably, although the concept of morphological awareness will be used for tasks that aim to assess conscious awareness of morphemes.

Interest in children’s and adults’ morphological knowledge emerged many decades ago (see for example, Chomsky, 1970); however, in the last 10–15 years, research in this area has burgeoned. Several meta-analyses have shown that morphological awareness is related to word reading, spelling, vocabulary development and (to a lesser extent) reading comprehension (Bowers, Kirby & Deacon, 2010; Goodwin & Ahn, 2010, 2013, see also Carlisle, 2010 for a review).

Learning to read builds on the oral language skills that children bring with them to school. Although studies have shown that morphological awareness is related to reading, the role of morphology in reading and learning to read is not well understood (Carlisle & Goodwin, 2013). Phonological, morphological, semantic and orthographic processing are at work in the process of reading, but the corresponding roles of these processes have not been addressed in reading research until recently. The links between phonological and orthographic abilities and between letter order and orthographic identification have been important in a number of reading models. A recent model of reading (Frost, 2012) criticises computational models of orthographic processing for not considering the word’s (and the reader’s) full linguistic environment. Understanding a written word depends on many processes beyond translating orthographic inputs into phonological outputs. Both phonological and semantic factors affect orthographic form, and Frost claims that “the representation of morphological information takes precedence over the representation of detailed phonological information in regard to the evolution of writing systems. Given that, it also takes precedence in regard to the cognitive processing of the orthographic structure by skilled readers” (p. 277). He further claims that native speakers notice the connections between spoken and written language through simple, implicit, statistical learning and by explicitly learning to spell. Both phonological and morphological information are important for orthographic processing, and Frost provides theoretical and empirical support for this claim. Frost’s model of word identification asserts that the most important correspondences between written and spoken language occur at the morphemic level. Even, though, morphemes have a phonological structure that may change from word to word, they have an orthographic form that is constant, and they convey meaning. Thus, morphemic awareness may support the automaticity of the word identification process. Furthermore, the lexical quality hypothesis supports the view that word meanings play an important role in written word identification (Perfetti, 2007). Morphology’s role in facilitating word recognition may represent one basis for the relationship between morphological abilities and reading comprehension. Morphological knowledge may also contribute to how new words are interpreted and learned in both spoken and written forms (Anglin, 1993; Bowers et al., 2010). Children develop morphological knowledge as a natural part of learning language (Carlisle, 2010). A child who knows the words “happy” and “unhappy” might discover that it is the “un-” at the beginning of “unhappy” that makes this word the opposite of “happy”, but a child who knows the words “happy” and “unhappy”, “finished” and “unfinished”, “pack” and “unpack”, among others, will be more likely to discover the meaning of “un-”. The ability to identify affixes and to understand their meanings may therefore facilitate vocabulary development (see Bowers & Kirby, 2010). Such learning and generalisation may occur in preschool, but learning to read is likely to enhance both morphological awareness and vocabulary development. In short, morphological abilities may contribute to reading comprehension via both word identification and improved vocabulary knowledge. However, the relationships between morphological and syntactic awareness and sentence and passage reading comprehension are not fully understood (see Tong, Deacon, & Cain, 2014).

The strongest evidence for a possible causal influence of morphological awareness on reading development comes from training studies. In one of the first such studies, Henry (1993) gave fourth and fifth graders explicit instruction about how words (with Anglo-Saxon, Latin and Greek morphological elements) were built from morphemes and about the meanings of these morphemes. The children who received decoding instruction based on word structure and word origin made significant gains in word structure knowledge and in decoding and spelling compared with untreated controls.

The effects of morphological awareness training have also been found in more recent training studies examining reading accuracy and reading comprehension skills in both typically developing children (see Bowers et al., 2010; Carlisle, 2010; Goodwin & Ahn, 2010, 2013; Reed, 2008 for reviews) and children with literacy difficulties (Arnbak & Elbro, 2000; Bowers et al., 2010; Goodwin & Ahn, 2010); however, there is a need for further evidence supporting the effectiveness of morphological training for improving reading skills. A particularly critical issue involves establishing how durable the effects of such training are (see Carlisle, 2010, for a discussion).

Norwegian is an Indo-European language belonging to the Germanic subgroup. As in English, the Norwegian writing system is morpho-phonemic, and the Norwegian language and orthography contain many of the prefixes, suffixes and derivations that are found in English (e.g., *un*lucky–*u*lykkelig, *mis*understand–*mis*forstå, fish/fisher/fishing–fisk/fisker/fisk*ing*). However, the pronunciation of morphemes in Norwegian is much more consistent than in English. Norwegian also contains many compound words consisting of 2, 3, 4 or even more words (brannbilutstyrboksen = the fire engine equipment box; skolehelsetjenesten = the school health service). For Norwegian children, it is important to “see the words in a word” to develop adequate reading speed and to understand that the final unit in compound words carries the main meaning. Given the structure of the Norwegian writing system, morphological knowledge appears likely to be an important contributor to reading development (Lyster, 2010), especially in later grades. Few studies have investigated the role of morphology in developing Norwegian language reading skills (but see Lyster, 2002).

The primary aim of the present study is to evaluate the long-term effects of preschool morphological awareness training on reading comprehension 6 years later. We present follow-up data from children who participated in an earlier intervention study examining the effects of morphological and phonological awareness training on reading development. The earlier study showed that phonological and morphological awareness training improved phonological and morphological awareness in preschool and reading ability in Grade 1, however, the morphological group showed greater gains on speeded word reading than the phonological group. Here we assess whether these effects persist over the long-term. Interpreting differences between the training groups and the control group is difficult since the control group was not formed by random assignment as were the two experimental groups, our analyses, however, control for differences between the groups at pretest.

Because of the regularity of the Norwegian orthography and the fact that school instruction teaches all children how graphemes represent sounds, we believed that the effect of phonological awareness training would be unlikely to persist in the present long-term follow-up. However, a comparison between the morphological awareness and phonological awareness training groups is important to determine the specificity of any training effects. Since both phonological and morphological training had effects on the children’s early linguistic awareness and written language abilities (Lyster, 2002), any long-term differences between these groups may be difficult to discover. Here we first assess whether either of the intervention groups show advantages over the control group in word reading and reading comprehension at a delayed (6 year) follow up. We also assess possible differences in word reading and reading comprehension at a 1 year follow-up to see if these variables act as mediators. Because both experimental groups received equivalent amounts of training from their preschool teachers and because all of the children’s school reading instruction programmes during Grade 1 were essentially identical (meaning that the main focus for reading instruction was on teaching the children the alphabetic principle and teaching them to decode by translating graphemes into phonemes, differences in long-term literacy outcomes between the experimental groups relative to the control group will indicate whether the effects are method-specific. Secondly we assess possible differences between the two experimental groups but are aware of the difficulties in interpreting differences between the training groups and the control group since the control group was not formed by random assignment. We address these difficulties by controlling for a range of variables at t1 known to be related to children’s reading comprehension (Mother’s educational level, vocabulary, phonological awareness and non-verbal ability).

## Methods

### Design and procedure of the preschool study

In their last preschool year (age 5–6), the children received either phonological awareness (phonology group, N = 106) or morphological awareness (morphology group, N = 127) training or were assigned to a control group (N = 36) that participated in ordinary preschool activities.

The duration of the training sessions in the experimental groups was approximately 30 min per week for 17 weeks. Occasionally, the training sessions were divided into 2 shorter periods of 15 min each and were conducted on 2 different days.

### The sampling procedure

Only Norwegian-speaking children and children with no serious developmental disabilities were included in the study. Teachers from each of the 25 preschool groups were randomly assigned to the two experimental groups. It was a challenge to find preschool teachers for a control group because the head of the municipal preschool system wanted all of the preschools to participate when we asked for permission for the study. However, before the preschool teachers could be accepted for participation, they needed to confirm that they could attend all of the training sessions and follow-ups after the intervention. For various reasons, for example supplementary training courses that had to be fulfilled during the intervention period, 5 preschool teachers were not available when the training for the intervention study occurred; thus, their groups, totalling 36 children, were selected to represent the control group. When the preschool teachers in the control group were absent, substitute teachers replaced them. All preschool teachers were qualified and had been in the community for at least 4 years and had attended the same training courses. The experimental group teachers attended several phonological awareness courses or morphological awareness courses (depending on their group assignment) before the intervention began and four courses during the intervention. The preschool teachers in the phonological group were introduced to phonological elements and phonological awareness activities, and those in the morphological group were introduced to morphological elements that are common in the vocabulary of 6-year-old children and to morphological awareness activities. Each of these meetings or courses introduced activities for the next 3–4 weeks of school, and meetings or courses were held on a monthly basis until the intervention period ended. The training was conducted on a whole-class basis or in groups of 6–12 children. For the largest groups, teacher assistants were present. These assistants primarily helped the children understand the tasks presented or helped to ensure that the children were attentive.

### The preschool intervention

#### Phonological awareness training

The phonological awareness training programme was similar to that used by Lundberg, Frost, and Petersen (1988) and included some of the phonological activities used by Bradley and Bryant (1983). The children participated in play-like activities involving syllable and sound blending and matching words that rhymed or started with the same sound. The most demanding activities in the phonological awareness programme were carefully introduced half way through the intervention period and involved identifying phonemes (What sound do you hear at the beginning of/sun/?) and sound deletion and manipulation activities (What remains if you delete/s/at the beginning of/sun/?). Sound identification and deletion activities involved, to start with, only words starting with continuant consonants so to make the sounds easier to identify.

#### Morphological awareness training

The morphological awareness training was similar in structure and activities to the phonological training, except that morphemes were the word segments targeted.

The morphological training involved teaching children to identify suffixes and prefixes in words and to recognise component words in compound words. The most demanding activities in the programme involved manipulating morphemes. One activity with compounds required the child to identify the two words in “skoeske” (shoe box), then delete the first word to leave only “eske” (box), and then delete “eske” to leave only “sko” (shoe). As the last step in this activity, the children were asked to change the sequence of the morphemes to create a new word “eskesko” (box shoe) and to indicate whether this was a real word. In this case, the resulting “eskesko” is not a Norwegian word. For other training items, such morpheme sequence changes would create a real Norwegian word.

A similar procedure was used for prefixes and suffixes. Inflections with the plural form –er (gutt–gutt*er*/boy–boys), the past-tense endings –*t* and –*te* (hoppe–hopp*et*/jump–jumped and smile–smil*te*/smile–smiled), the comparative and superlative forms of adjectives and adverbs (blå–blå*ere*–blå*est*/blue–bluer–bluest and rask–rask*ere*–rask*est*/fast–faster–fastest) and derivations (happy/unhappy) were used in the training. For example, the children would be asked about the word “lykkelig” (happy), and if they knew the meaning of the word, they were then asked to add “u” (un-) in front and determine the meaning of the new word. They were then asked about the meaning of “*u*lykkelig” (unhappy), how it differed from “lykkelig” in form and meaning and whether they knew other words that could be changed in the same way to yield the opposite meaning. The same procedure was used for verb endings (regular verbs only) and regular nouns to help the children identify changes in verb endings and in the plural form of regular nouns. For example, the children made figures to use in the training. One of these figures they called “pei” (a nonword with an adequate Norwegian orthographic structure). This “pei” (a drawn figure) was placed on a flannel board, and the children placed the name “pei” beside it. Because the “pei” was “trist” (sad), we also added the word “sad” next to it and talked about what sad means; we also mentioned the synonym “unhappy” and the antonym “happy” on this occasion. Another “pei” was then added, and the children were asked whether it is correct to say “pei” when there were two of these figures. The dialogue with the children ended with an agreement that there were two “pei-er” (peis) and that the written word, like the spoken word, needed an ending (the inflection –er was added to “pei” on the flannel board) (see also Author). Comparatives and superlatives were also added in this manner. The second “pei” appeared to be even sadder than the first one; the second “pei” was “tristere” (sadder). Actually, he was the “tristest” (saddest) of the two. For the comparatives and superlatives, the children were also shown that the written words were changed in accordance with changes in the spoken words.

#### Exposure to print in the experimental groups and the control group

In Norway, there is no systematic teaching of letters and no reading instruction before children enter school. The Norwegian preschool is not a part of the school system. There is no reading instruction, even in the last year before school entrance. Children have books read to them and activities are used to stimulate their language development but a large part of the time is devoted to play activities.

However, there is some teaching of letters and written words, especially with children’s names and play writing. Often, words are written on posters or children’s drawings that have been placed on the walls, providing some exposure to print during the preschool stage.

In both the phonological and morphological awareness programmes, the children were exposed to print for some activities (to the same extent in both programmes according to our instructions and according to what we observed when visiting and observing teaching), but most of the phonological or morphological training sessions contained little print exposure. For example, the children in the phonological programme listened to words and observed in print that words with the same rime have endings that look alike. The children also listened to the sounds at the beginning of words such as “sol” (sun), “seil” (sail), and “sekk” (sack); when shown the written words, the children saw that the first sound in each word was also written in the same way, with the letter “s”. The children in the morphological programme observed in print how two words could make a new, longer word and, as presented above, they saw how prefixes and suffixes were added to and deleted from words. Instead of focusing on the initial sounds of words, they focused on the suffixes and instead of focusing on the rime, they focused on the affixes. The control group had some exposure to print during their ordinary activities but they received no deliberate teaching concerning the phonological or morphological form of words.

### Participants in the follow-up study

Of the 269 children in the original study, 115 (22 from phonological group, 63 from the morphological group and 30 from the control group) were included in the follow-up to be tested in Grade 6 using a word reading test and 3 text reading tests. The children attended 18 different schools after preschool, but only 9 classes in 8 different schools were followed up in Grade 6. There were no differences between the schools who remained in the study and those that left as far as national reading results are concerned. The loss of children at the follow-up and the lack of balance between the group sizes raise the possibility that the outcome of this study will be biased. However, Little’s Missing Completely at Random (MCAR) test of all the variables indicated that the children in the follow-up sample did not differ from a random sample of the 269 children tested at Time 1, χ^2^ (100) = 117.93, *p* = .066. In addition, we measured several pre-intervention factors (the children’s cognitive level, their mothers’ educational level, and the children’s vocabulary and preschool phonological abilities) that are known to influence reading development. On none of these pre-intervention factors did the children in the follow-up sample differ at Time 1 from the children that were not followed up. The *p* values for the *t* tests were: .46 for the children’s cognitive level, .79 for their mother’s education, and .16 for the children’s vocabulary and .25 for the preschool phonological abilities composite. Also, comparing the reading level for all children in grade 6 for the schools included on the measures used in this follow-up, there were no statistically significant differences between the schools on any of the reading tests. For Word Reading, F (7,117) = 1.958, *p* = .074 and for the three text reading measures F (7,117) = 1.74, *p* = .107, F (7,117) = .895, *p* = .513 and F (7,117) = 1.674, *p* = .122, respectively.

The main approach to reading instruction in all of the schools in Grade 1 was a phonics approach with a clear focus on the alphabetic principle and on phonological strategies for decoding words (a grapheme-to-phoneme strategy). The various school classrooms received children from a large number of different preschools. However, most of the children stayed in the same classroom throughout their first 6 years of school. All school teachers had a 4 year teacher-education and had been in the same school for at least 4 years. Working in the same community meant that they attended the same continuing education courses and got the same teaching support from the community’s school service program.

### Measures

#### Preschool and early Grade 1 measures

The following linguistic measures and information about mother’s education were collected before the intervention in the beginning of the last preschool year. Non-verbal IQ and vocabulary measures were collected early in Grade 1 and were used as additional control variables in the analyses reported below.

##### Initial phoneme matching

The children were presented with a row of three pictures and were asked to select the picture that started with the same sound that the tester pronounced (for example find the picture of a sun when the question was to find an item starting with/s/). Two practice items and 10 test items were given. Both consonants and vowels were used as target phonemes.

##### Phoneme blending

For each item, the children were presented with a row of three pictures. The phonemes in the target word were pronounced with an interval of approximately 1*/*2 s between them. The children were asked to mark the picture that matched the resulting word (for example finding the picture of a man when given the sounds/m/-/a/-/n/). The length of the words varied from two to four sounds. Two practice trials and nine test items were presented.

##### Phoneme counting

Each word was presented orally together with an easily recognisable picture. The children’s task was to count the phonemes in the word and to mark each phoneme by making a pencil stroke in an empty box next to the picture, The tester would say the word slowly and ask, for example, how many sound they heard in the word “cat” as well as telling the children to make one pencil stroke for each sound. The children were given one practice trial and six test items.

##### Deletion of initial phonemes

For each item, the children were presented with a row of three pictures. A word was presented, and the children were told that if the first sound of the word was deleted, then one of the pictures in the row would match the resulting word (e.g., What is left if you delete/take away the first sound/r/in rice?). Two practice items were presented, followed by 10 test items. The children were presented with words with both CV and CCV onsets.

##### Mothers’ educational level

The mothers reported their years of education and type of education. Their total number of years in school/education from Grade 1 is reported.

##### Nonverbal IQ

Data for Raven’s Progressive Matrices (Raven, Raven, and Court, 1988) were collected in Grade 1. This test is a nonverbal group test consisting of 60 multiple choice questions. For each test item, the subject is asked to identify the missing item that completes a pattern.

##### Vocabulary

Vocabulary data were collected in the beginning of Grade 1 using the WISC-R [Norwegian version (Undheim, 1978)]. In this task, the children were asked to explain the meanings of the presented words (e.g., umbrella: can you explain the word umbrella). Children were given 1 point for a partly correct answer and 2 points for a well defined explanation of the word. It should be noted that this task was conducted after the intervention.

#### Grade 1 reading tests

Grade 1 reading data were collected at the end of Grade 1. We used Gjessing’s (1958) standardized test for word reading and text reading which has been widely used in Norway. Both the word reading and the text reading tasks are silent reading tasks.

##### Word reading

For each item in the word reading test the children were presented with an easily recognized picture and a varying number (4–8) of words to match the picture. The children had to mark the word corresponding to the picture. There were 3 trial items and 36 test items. Children completed as many items as they could in four and a half minutes. The test–retest reliability is reported to be 0.87 for the total word and text reading tasks.

##### Text reading

The children were presented with passages of increasing length. After each passage there is one question to be answered by putting a cross on a picture representing the correct answer. There were 19 test items. Children completed as many items as they could in 12 min. Each correct answer gave a score of two points.

#### Grade 6 reading tests

Grade 6 reading data were collected at the end of grade 6. These reading tests are part of a standardised Norwegian reading assessment battery (Nasjonalt læremiddelsenter, 1995). There is a tendency for ceiling effects for all subtests because the battery was developed to identify struggling readers. However, because this test is the only standardised Norwegian battery available for this grade and because reliable self-made tests would be impossible to develop within the limited amount of time we had to select tests and conduct the testing, we decided to use the existing test battery.

##### Word reading

This test was a silent word reading task similar to the one given in Grade 1. For each item the children were presented with a word followed by 4 easily recognized pictures and asked to select the picture that represented the word. The test included 40 items and the children completed as many items as they could in 3 min. The score was number of words correct.

##### Continuous, narrative text reading

This relatively challenging, narrative text contained 1099 words. The reading task was followed by 16 multiple choice questions asking for facts and information given in the text, each of which had four possible responses for the children to mark. Children were allowed 8 min to read the text and answer the questions. The text remained visible while the children answered the questions.

##### Discontinuous expository text reading 1 (text and table)

This task consisted of a relatively short text and a table with information about proteins, fat and carbohydrates in various foods. This task is referred to as discontinuous because children had to go back and forth between the text and the table and the questions to find answers. Seven multiple choice questions with three possible answers each were presented. The answers to the questions were primarily found in the table, but the text also included information about the task. The children were given 3 min to answer as many questions as possible.

##### Discontinuous expository text reading 2 (text and map)

This task included a short text accompanied by a map of the world with written information on the map. Bars were placed at different places on the map to show the amount of production in each area. Some of the bars were grey (showing the amount of oil produced), and the remainder of the bars were white (showing the amount of gas produced). As for the first discontinuous test, the children here had to go back and forth between the text, the map and the questions to find answers to the questions. The answers to the questions were found primarily on the map with the bars but were also supported by the text. The children were given 2 min to answer as many questions as possible.

## Results

Table 1 shows the descriptive statistics and reliabilities for the relevant variables for the three groups at preschool and early Grade 1 (two control variables), end of Grade 1 and Grade 6.

**Table 1:** Means, standard deviations, and reliabilities for the preschool, Grades 1 and 6 measures

Measures	Max score	Phonology M (SD)	Morphology M (SD)	Control M (SD)	Alpha
Age in months t1	–	75.40 (3.59)	75.91 (3.22)	77.17 (3.13)	–
Pre-school measures					
Mother’s education^a^	–	12.11 (2.12)	11.55 (2.13)	10.80 (1.92)	–
Identifying first phoneme	10	8.50 (1.69)	8.11 (2.02)	8.03 (2.17)	.67
Phoneme blending	9	7.05 (1.59)	6.91 (1.98)	7.20 (1.61)	.53
Counting phonemes	6	2.67 (1.66)	1.86 (1.72)	2.38 (1.23)	.61
Phoneme deletion	9	4.95 (2.23)	4.80 (1.98)	4.94 (2.03)	.61
Vocabulary^c^	66	9.68 (2.59)	9.30 (2.45)	9.73 (3.33)	.81^b^
Raven’s PM^c^	60	23.82 (6.82)	22.75 (7.44)	21.88 (5.72)	–
End of Grade 1 measures					
Word reading	36	21.96 (8.44)	23.58 (8.81)	21.64 (7.30)	.87
Text reading	38	20.88 (11.18)	20.57 (11.68)	19.22 (10.30)	.87
Grade 6 measures					
Word reading	40	37.82 (4.10)	37.38 (4.78)	36.43 (4.28)	.97
Plain text	16	11.23 (6.19)	12.83 (4.52)	10.50 (5.64)	.97
Text and map	8	6.59 (1.76)	6.81 (1.38)	5.57 (2.57)	.83
Text and table	9	8.00 (1.38)	7.76 (1.61)	6.97 (2.22)	.83

^a^Years of education

^b^The reliability measure, the Spearman Brown formula, is taken from the Norwegian standardisation of the WISC-R

^c^Early Grade 1 measure

Notably, at preschool, there were some imbalances between the groups on key background measures. The groups did not differ in terms of WISC vocabulary (F = .77, *p* = .462), Nonverbal IQ (F = .121, *p* = .299), first phoneme identification (F = 1.47, *p* = .232), phoneme blending (F = .421, *p* = .657) and phoneme deletion (F = .170, *p* = .844); however, the two intervention groups had higher values for mother’s educational level than the control group (F = 5.395, *p* = .005). The phonological awareness training group also scored significantly higher than the morphological training group on counting phonemes (F = 6.524, *p* = .002).

### Long-term effects of the training

Two structural equation models were estimated to test whether phoneme and morpheme training in kindergarten affected word reading and reading comprehension 1 year as well as 6 years later. In these models, we used maximum likelihoods with information from all 269 participants. Two dummy variables were used to contrast each intervention group with the control group [one variable contrasting the phoneme training (1) with the control group (0) and the other contrasting the morphological group (1) with the control group (0)]. Because both Little’s MCAR test and the series of *t* tests indicated that the longitudinal follow-up sample did not differ significantly from the original 269 participants, full information maximum likelihood was considered to be the most powerful method for handling the missing values (Enders, 2010).

**Fig. 1:**
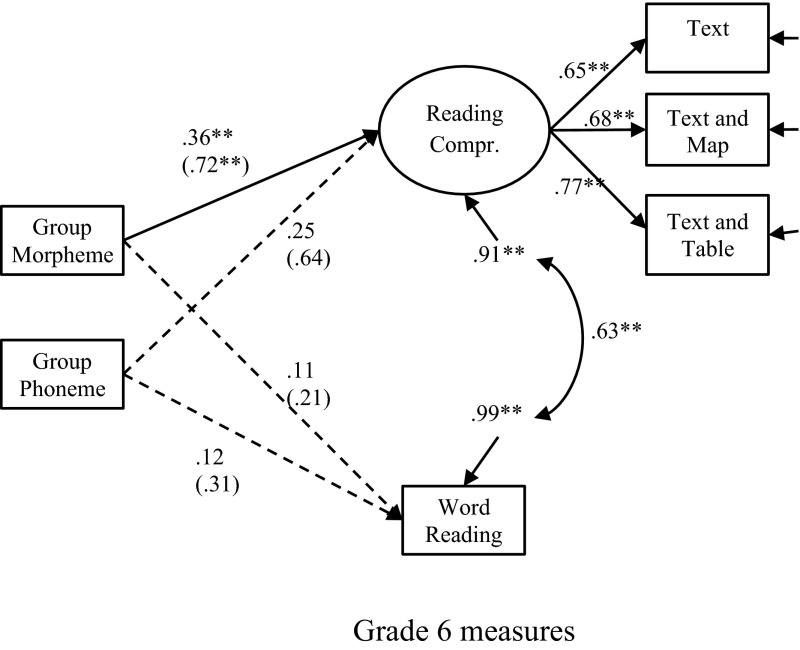
The long-term effects of phoneme awareness and morphological training in kindergarten on reading comprehension and word decoding in Grade 6. Latent variables are drawn as *ellipses*, and observed variables are drawn as *rectangles*. *One*-*headed arrows* are regressions, factor loadings or residuals, and *two*-*headed arrows* are correlations. *Solid lines* represent significant effects, and *dashed lines* represent non-significant effects. Coefficients outside of brackets are fully standardised on both the X and Y variables. Coefficients within brackets are standardised only on the Y variables, indicating the difference between the training groups and the controls in standard deviation units on the dependent variable. **p < .01

In the first model (see Fig. 1), only the dummy variables and the reading variables measured at Grade 6 were included. The children who received morpheme training in preschool showed significantly better reading comprehension, but not word reading skills in Grade 6 compared with the control group. The effect size for morpheme training on reading comprehension was .72, *p* = .002 (y-standardized coefficients, can be interpreted as Cohen’s d). The small sample size for the control group may lead to bias in effect sizes. However, Hedge’s g shows the same effect size as our y-standardized coefficient (.717). Also, the effect size is unexpectedly high. It may be overestimated because the ceiling effects in the Grade 6 measure produced smaller variance estimates. The children who received phoneme awareness training in kindergarten did not differ from the controls in either of the reading measures in sixth grade. The latent reading comprehension construct consisted of three variables: text reading, text and map reading, and text and table reading. Word reading was estimated as an observed variable. The dummy variables reflecting the training groups explained 9.4 % of the variation in reading comprehension skills 6 years after the training occurred. The model’s fit was excellent: χ^2^ (6) = 7.62, *p* = .267, RMSEA = .048 (CI 90 % .00–.137), CFI = .986, SRMS = .035. A comparable model with the phoneme training group as the reference group (contrasting phoneme training to morpheme training) showed that the effect of the morpheme intervention did not differ significantly from the effect of the phoneme intervention, effect size = .08, *p* = .785.

**Fig. 2:**
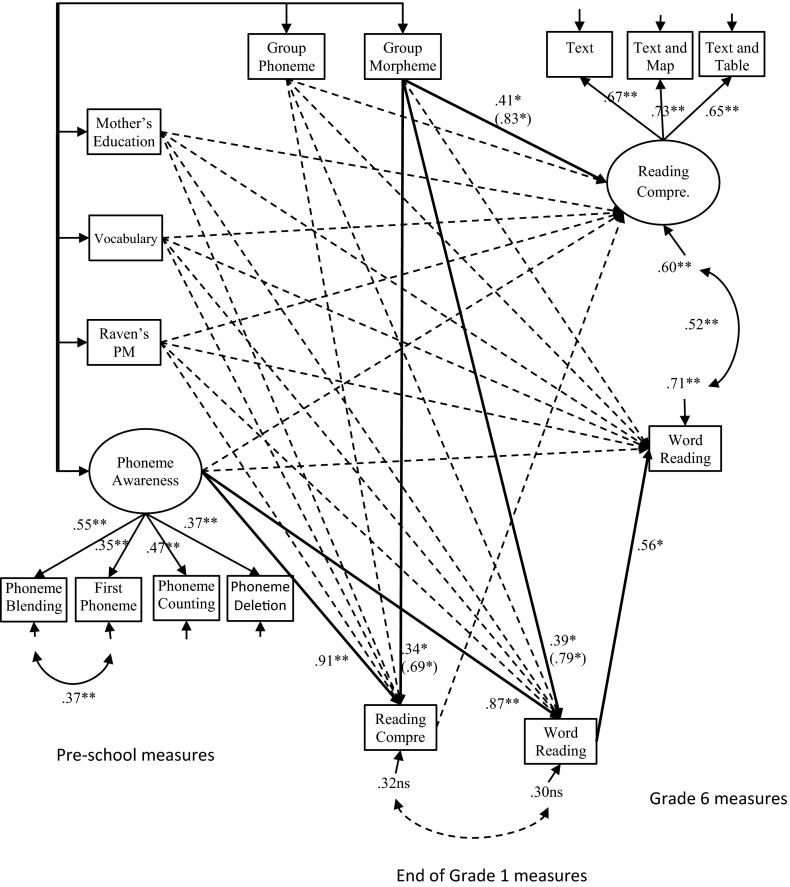
The long-term effects of phoneme awareness and morphological training in kindergarten on reading comprehension and word decoding in end of Grades 1 and 6, controlling for differences in preschool cognitive skills and variations in mother’s education. Latent variables are drawn as *ellipses*, and observed variables are drawn as *rectangles*. *One*-*headed arrows* are regressions, factor loadings or residuals, and *two*-*headed arrows* are correlations. *Solid lines* represent significant effects, and *dashed lines* represent non-significant effects. Coefficients outside brackets are fully standardised on both the X and Y variables. Coefficients within brackets are standardised only on the Y variables, indicating the difference between the training groups and the controls in standard deviation units on the dependent variable. **p < .01; *p < .05

The positive effect of morpheme training in kindergarten on reading comprehension in Grade 6 was also confirmed when the preschool differences in mother’s education, vocabulary, Raven’s nonverbal abilities and phoneme awareness were controlled in a second model. In this model, we also included reading comprehension and word reading at Grade 1 to see if any effect of the interventions on Grade 6 reading comprehension and word reading was mediated through reading comprehension and word reading respectively at Grade 1. This model is shown in Fig. 2 and fit the data very well: χ^2^ (54) = 66.31, *p* = .121, RMSEA = .029 (CI 90 % .00–.051), CFI = .984, SRMS = .049. In this model the residuals for phoneme blending and first phoneme are correlated (because the phoneme awareness variables were used as controls (and dimensionality was not a research question), this modification was considered justifiable). In this model, both phoneme awareness and reading comprehension were estimated as latent variables, and the other variables were observed variables (including the two dummies).

In this model the morpheme trained group showed significantly better reading comprehension skills at Grade 6 than the control group. The effect size for morpheme training on reading comprehension was .83, *p* = .031 (Hedge’s g = .826). The morpheme intervention also improved both reading comprehension (effect size = .69, *p* = .030) and word reading (effect size = .79, *p* = .012) at the end of Grade 1 compared to the control group. For these last effect sizes Hedge’s g was similar in size. The effect of morpheme training on reading comprehension at Grade 6 was however, not mediated through reading comprehension at Grade 1, effect size = .11, *p* = .539. In addition, we can observe that the mothers of the children in the phoneme awareness training group were more educated than the mothers of the control children and that the children in the morpheme training group originally had lower phoneme awareness skills at pre-test compared with the control group children. A total of 40 % of the variance in reading comprehension and 29 % of the variance in word decoding was explained by the training groups and the control variables together. A comparable model with the phoneme training group as the reference group showed that the morpheme intervention did not differ significantly from the effect of the phoneme intervention (effect size = .19, *p* = .231).

## Discussion

This study examined the long-term effects of a morphological and a phonological awareness training programme delivered in preschool on later reading abilities. Our main focus was on the development of reading comprehension and we found that morphological training produced significantly better reading comprehension than in the control group at Grade 1 as well as Grade 6. In contrast the parallel phonological awareness programme produced no durable effects on reading comprehension. We also found effects of the morphological training but not the phonological training on word reading at Grade 1. There was no long-term effect on word reading at Grade 6 for the morphological preschool intervention. However, there was a ceiling effect on the word reading task at Grade 6 which may have served to conceal any genuine effects. In addition the t1 variables included in the model may also have captured variation in students’ word reading skills as well as reading comprehension skills at Grade 1 and reduced the contribution of these skills on reading comprehension at Grade 6. Lack of mediation from the morphological training through Grade 1 reading comprehension may reflect the large differences between the one easy Grade 1 reading comprehension measure and the far more complicated measures used in Grade 6 which included many morphologically complex words. Although several studies have shown both direct and indirect mediating effects from morphological awareness to reading comprehension through word reading (e.g., Deacon, Kieffer, & Laroche, 2014; Perfetti, Landi, & Oakhill, 2005), other studies have not shown such mediating effects. Kieffer and Lesaux’s (2012b) study of the relationship between morphological awareness and reading comprehension in Grade 6 English speaking children and several groups of English language learners found a direct contribution of morphological awareness on reading comprehension and no mediation through word reading fluency (see also Kirby et al., 2012). Our finding is also in line with Nagy’s (2007) assumption that the ability to extract semantic and syntactic information from morphologically complex words plays an important role in real-time comprehension processing over and above the role such ability has in earlier language acquisition.

Our results support earlier findings concerning the effect of morphological awareness training on reading comprehension (e.g., Bowers et al., 2010; Goodwin and Ahn, 2010). When they entered school and learned to read, all of the children in the present study were involved in a reading programme with a strong focus on the alphabetic principle and grapheme–phoneme correspondences. Children from the morphological group, however, brought with them additional knowledge about word meaning and form that they might have applied when learning to read, especially when reading more demanding texts.

The results of the present study are unique because they show the long-term effects of morphological training on reading comprehension skills over a 6-year period. Few intervention studies provide long-term follow-up data (Suggate, 2014) and no other studies have looked into effects of morphological interventions over such a long period.

Carlisle (2010) states that “more needs to be done to provide a clearer understanding of how, when and why morphological awareness instruction contributes to students’ literacy development” (p. 464). Because the training in the present study was broadly based, we cannot identify specific components of the training that may have been particularly critical. It does appear, however, that morphological training in preschool has effects on the very early development of reading in Grade 1 as well as long-term effects on reading development. Our findings also are in line with Brinchmann, Hjetland, and Lyster (2015) who found that a broad based vocabulary intervention seems to be effective not only for understanding unknown words with known morphemes but also for reading comprehension. Given the findings in the present follow-up study we suggest that the morphological focus, working with form and content “hand in hand” resulted in a cumulative effect of the morphological training and word learning. Understanding the meanings of “disadvantage, disagreement and disappointment” and being aware of the meaning of the prefix dis-, may help a child understand a new word such as disobedient, even if only the word “obedient” was known before.

Also, both the strong focus on word compounds and the introduction of relatively few affixes in a large number of words may have enhanced learning from a statistical perspective and improved children’s understanding of how morphemes work as building blocks in words. Some studies have provided students with morphological awareness strategies to help them understand the meaning of unknown words during reading (e.g., Baumann, Edwards, Boland, Olejnik, & Kame’enui, 2003), but few studies appear to have focused on word meaning and morpheme meaning as explicitly and intensely as we did in the present study. Additionally, Clarke, Snowling, Truelove, and Hulme (2010) show that broad-based oral language training has a significant effect on reading comprehension, both immediately and at follow-up. Our findings, however, indicate not only that morphology is important in the long term but also that *early* and *direct* introduction to the form and semantics of morphemes and to how words are built from morphemes is helpful.

The preschool teachers who participated in our study received relatively extensive training. Videos from their teaching show high fidelity; that is, the teachers knew the programme well and knew how to support all the children. Working within the “zone of proximal development” framework (Vygotsky, 1978), the teachers in all three groups appeared to be proficient in guiding the children towards an understanding of and a solution to a task or a question without presenting the answer themselves.

## Limitations

Several limitations of this study should be considered when interpreting the results. The primary limitations are the relatively small number of children in the phonology group in the follow-up-study and the small size of the control group. A second limitation, as mentioned above, is related to the Grade 6 reading measures. Ceiling effects limited our assessment of the full variability of the students’ reading abilities. Also, we attempted to assess the extent to which effects on reading may have been mediated by changes in morphological awareness at immediate post-test. However, because of strong ceiling effects for the morphological awareness measures at this time point, we did not include these measures in our model. Therefore we have not been able to investigate whether the effects of training on Grade 6 reading comprehension are mediated by the immediate post-test results in preschool. It is also important to be aware that the ceiling effects for the Grade 6 reading measures may have lead to larger standardized effect for the morphological intervention than would have otherwise occured.

## Implications for practice

The present study, along with earlier research, suggests that morphological awareness training may be an important part of early reading programmes alongside phonological awareness training. Although we must develop further knowledge of “how, when, and why morphological awareness instruction contributes to students’ literacy development” (Carlisle, 2010, p. 464), we know enough to include morphological awareness training as a valuable component of preschool language programmes (especially if school starts as late as age 6–7 years as it does in Norway) and early reading programmes. As Carlisle and Goodwin (2013) concluded and Nunes and Bryant (2006) noted, we cannot assume that students learn the complex morphological system by themselves. By giving preschoolers and young schoolchildren knowledge of the morphemic structure of words and helping them to integrate meaning with the spoken and (when they begin to read and write) written forms of words, we may hope to enhance their oral and written language development.
